# Welche Rolle spielen die soziale Herkunft und die Schulform für die Wahrnehmung von digitalem Feedback und das akademische Selbstkonzept während der COVID-19 Pandemie?

**DOI:** 10.1007/s35834-022-00335-9

**Published:** 2022-05-05

**Authors:** Fabian Alexander Emde, Kira Elena Weber

**Affiliations:** 1 Institut für Bildungswissenschaft, Leuphana Universität Lüneburg, Universitätsallee 1, 21335 Lüneburg, Deutschland; 2 Leibniz-Institut für die Pädagogik der Naturwissenschaften und Mathematik, Olshausenstraße 62, 24118 Kiel, Deutschland

**Keywords:** Akademisches Selbstkonzept, COVID-19, Feedback, Distanzunterricht, Schüler*innen, Soziale Ungleichheit, Distanzlernen, Academic self-concept, COVID-19, Feedback, Distance education, Students, Social inequality, Distance learning

## Abstract

Wie Schüler*innen Feedback wahrnehmen, bedingt die Wirkung von Feedback auf schulische Leistungen sowie die Genese von akademischen Selbstkonzepten. Durch die COVID-19 Pandemie veränderten sich die Rahmenbedingungen des schulischen Lernens und somit auch die Feedbackgelegenheiten im Unterricht. In der vorliegenden Studie wurde anhand einer Stichprobe von 668 Schüler*innen verschiedener Schulformen untersucht, wie häufig diese digitales Feedback von Lehrkräften erhielten und wie sie das Feedback wahrnahmen. Dabei wurde analysiert, welche Rolle die Schulform und die soziale Herkunft (sozioökonomischer Status und Migrationshintergrund) bei der Wahrnehmung von Feedback spielen und ob die soziale Herkunft, die Schulform und das wahrgenommene Feedback das akademische Selbstkonzept vorhersagen können. Bezogen auf die Schulform nahmen Schüler*innen an Gymnasien das Feedback ihrer Lehrkräfte als weniger fair wahr, akzeptierten es weniger und sahen weniger Nutzen in dem Feedback. Die soziale Herkunft von Schüler*innen mit und ohne Migrationshintergrund spielte keine Rolle bei der Wahrnehmung des Feedbacks. Die Ergebnisse eines multiplen Regressionsmodells zeigten, dass der sozioökonomische Status, die wahrgenommene *Fairness* und der *Nutzen* des Feedbacks positive Prädiktoren für das *akademische Selbstkonzept* im Distanzunterricht sind. Die vorliegende Studie liefert damit erste Hinweise auf die Wahrnehmung von digitalem Feedback und den Zusammenhang zum akademischen Selbstkonzept. Die Ergebnisse implizieren, dass Lehrkräfte die Wahrnehmung des Feedbacks durch die Schüler*innen stärker einbeziehen sollten, um insbesondere das akademische Selbstkonzept zu stärken.

## Abstract (en)

How students perceive feedback shapes the impact of feedback on academic achievement and the development of academic self-concepts. The COVID-19 pandemic changed the framework of school learning and thus the opportunities for feedback in the classroom. In the present study, a sample of 668 students from different types of schools was used to investigate how often they received digital feedback from teachers and how they perceived the feedback. We analysed the role of school type and social background (socioeconomic status and migration background) in the perception of feedback and whether social background, school type, and perceived feedback can predict academic self-concept. In terms of school type, students in the higher academic track (Gymnasium) perceived their teachers’ feedback as less fair, accepted it less, and saw less benefit in the feedback. The social background of students with and without a migration background did not play a significant role in their perception of the feedback. The results of a multiple regression model showed that socioeconomic status, perceived fairness, and usefulness of the feedback were positive predictors of academic self-concept in distance education. Thus, the present study provides preliminary evidence on the perception of digital feedback and its relationship to academic self-concept. The results imply that teachers should more strongly incorporate students’ perceptions of feedback to especially enhance academic self-concept.

## Einleitung

Feedback zählt zu den einflussreichsten Faktoren auf schulische Lernprozesse (Hattie 2009) und wirkt sich sowohl auf Motivation als auch auf Metakognition aus (siehe z. B. Narciss 2004). Im schulischen Unterricht kann Feedback als „postresponse information that is provided to a learner to inform the learner on his or her actual state of learning or performance“ (Narciss 2008, S. 127) verstanden werden. Forschungsergebnisse zur Wirksamkeit von Feedback (Kluger und DeNisi 1996) sind zum Teil widersprüchlich. Für Narciss (2014) deuten die inkonsistenten Befunde darauf hin, dass verschiedene situative und individuelle Faktoren im Zusammenhang mit Feedback berücksichtigt werden müssen. So hängt die Wirkung von Feedback unter anderem davon ab, wie Schüler*innen das Feedback wahrnehmen und verarbeiten (Strijbos et al. 2021). Auch Hattie und Wollenschläger (2014) weisen darauf hin, dass das Empfangen von Feedback der weitaus relevantere Teil bei der Erforschung von Feedback in Lehr-Lernprozessen ist. Entsprechend wird in aktuellen Studien die Relevanz des wahrgenommenen Feedbacks von Seiten der Schüler*innen für ihre Leistungen und ihr Verhalten unterstrichen (Schwab et al. 2022; Strijbos et al. 2021; Brooks et al. 2019).

Neben dem direkten Einfluss von Feedback auf die schulische Leistung und den Lernprozess von Lernenden (Hattie und Timperley 2007) spielt Feedback auch eine bedeutsame Rolle für die Genese von akademischen Selbstkonzepten (Blöte 1995; Chen et al. 2011), die wiederum reziprok mit Leistungen und Leistungsmotivation von Schüler*innen zusammenhängen (Hansford und Hattie 1982; Marsh et al. 2005). In Studien zum Zusammenhang von Feedback und dem akademischen Selbstkonzept wurde überwiegend das positive und negative Feedback untersucht (z. B. Nicaise et al. 2006; Chen et al. 2011), jedoch nicht das wahrgenommene Feedback.

Die bundesweiten Schulschließungen im März 2020 führten zu neuen Herausforderungen in der Gestaltung von Lehr-Lernprozessen und somit auch dem Feedback im Unterricht. Im Zusammenhang mit der COVID-19 Pandemie stellt sich daher die Frage, wie oft Schüler*innen Feedback von ihren Lehrkräften erhielten und wie sie das digitale Feedback wahrgenommen haben. Bisherige Studien konnten zeigen, dass der Großteil der Schüler*innen Rückmeldungen und Feedback von ihren Lehrkräften während der COVID-19 Pandemie erhielt (Lorenz et al. 2020; Wößmann et al. 2021). Diese Studien evaluierten jedoch vorrangig die Häufigkeit von Feedback und nicht das wahrgenommene Feedback von Seiten der Schüler*innen. Ebenso ist unklar, welche Rolle die soziale Herkunft bei der Wahrnehmung von digitalem Feedback im Distanzlernen spielt.

Die soziale Herkunft und der Migrationshintergrund von Schüler*innen stellen – neben den Schulmerkmalen – wichtige Einflussfaktoren für die schulische Kompetenzentwicklung dar, welche sich insbesondere im Rahmen von großen Schulleistungsstudien (z. B. PISA, IQB-Bildungstrends und TIMSS) zeigen (Weis et al. 2018; Mahler und Kölm 2018; Henschel et al. 2018; Stubbe et al. 2019). Schüler*innen mit einem niedrigen sozioökonomischen Status oder einem Migrationshintergrund stellen demnach eine Risikogruppe im deutschen Bildungssystem dar. Erste Befunde deuten darauf hin, dass die Auswirkungen der COVID-19-Pandemie diese bereits vorliegenden sozialen Disparitäten noch einmal zu verstärken scheinen (Engzell et al. 2021). Zusätzlich zu der sozialen Herkunft der Schüler*innen sind unterschiedliche Entwicklungschancen auch auf die Schulform zurückzuführen (Baumert et al. 2006). Schulformunterschiede werden durch die Zusammensetzung der Schüler*innenschaft und unterschiedliche Lehrpläne und Unterrichtskulturen erzeugt.

Entsprechend fokussiert die vorliegende Studie zum einen darauf wie die Schulform, der sozioökonomische Status und der Migrationshintergrund von Schüler*innen die* Wahrnehmung der Feedbackhäufigkeit, *die *wahrgenommene Fairness, den Nutzen und die Akzeptanz des digitalen Feedbacks* und das *akademischen Selbstkonzept* bedingen. Zum anderen wird untersucht, ob die soziale Herkunft, die Schulform, die *Feedbackhäufigkeit*, die *Fairness*, der *Nutzen* und die *Akzeptanz* des wahrgenommenen Feedbacks Prädiktoren für das akademische Selbstkonzept sind.

### Feedback im schulischen Kontext

Im schulischen Kontext dient Feedback dem Ziel die Diskrepanz zwischen der gegenwärtigen Leistung und dem gewünschten Lernziel zu verringern (Hattie und Timperley 2007) und gilt daher als wichtiger Bestandteil von Unterrichtqualität (Helmke 2021). Jedoch ist Feedback nicht per se lernförderlich (Kluger und DeNisi 1996; Shute 2008). Sowohl Strijbos et al. (2021) als auch Hattie und Timperley (2007) betonen, dass die Effektstärken von Feedback auf Lernleistungen von verschiedenen Variablen und Kontextfaktoren abhängig sind. Zum Beispiel ist das Verhältnis von *Feedbackhäufigkeit* und Lernzuwachs nur zu einem gewissen Grad proportional (Kluger und DeNisi 1996; Lam et al. 2011). Gleichwohl spielt der quantitative Faktor – also die *Feedbackhäufigkeit* – eine wichtige Rolle im Feedbackprozess.

In dem Feedback Modell von Narciss (2006) wird das Zusammenwirken der individuellen Faktoren und die inhaltliche und formale Qualität des externen Feedbacks in Lehr/-Lernsettings veranschaulicht (siehe auch Narciss 2014). In solchen multidimensionalen Perspektiven des Feedbacks werden Schüler*innen als aktive Akteure*innen angesehen, die Feedback aufnehmen, interpretieren und anwenden (Brooks et al. 2019). Der Wahrnehmung von Feedback wird demnach eine entscheidende Bedeutung beigemessen. Nach Strijbos und Müller (2014) sind dabei unter anderem personale Aspekte (z. B. Geschlecht, sozioökonomischer Status, Migrationshintergrund) bei der Verarbeitung von Feedback bedeutsam, ebenso wie der Rahmen (Kontext) des Feedbacks. Institutionelle Unterschiede (z. B. Lehrpläne, Stundentafeln und Unterrichtskultur) und Kompositionseffekte (Zusammensetzung der Schüler*innenschaft) machen den Kontext des Feedbacks in Schule aus (Baumert et al. 2006). Demzufolge können Unterschiede in der Wahrnehmung des Feedbacks von Schüler*innen auch auf die Schulform zurückgeführt werden. In einem Review zum Fernunterricht während der COVID-19 Pandemie (Helm et al. 2021) konnten jedoch keine Unterschiede hinsichtlich des Feedbacks nach Schulform festgestellt werden.

### Einfluss personaler Faktoren auf die Wahrnehmung von Feedback

Im Hinblick auf den Einfluss personaler Faktoren (z. B. soziökonomischer Status und Migrationshintergrund) bei der Wahrnehmung von Feedback, untersuchte Sortkær (2019) in einer Studie (*n* = 17.283 Schüler*innen) den Zusammenhang zwischen dem sozioökonomischen Status von Schüler*innen und dem wahrgenommenen Feedback der Lehrkräfte in Dänemark, Finnland, Norwegen, Island und Schweden. Die Ergebnisse der Studie zeigen, dass Schüler*innen mit einem niedrigeren sozioökonomischen Status mehr directive feedback und Schüler*innen mit einem höheren sozioökonomischen Status mehr facilitative feedback wahrnahmen. Dass Unterschiede in der Wahrnehmung von Feedback nach dem sozioökonomischen Status von Schüler*innen eher eine Ausnahme darstellen, konnten Eryilmaz und Sandoval-Hernández (2021) anhand von Daten aus 75 Ländern zeigen. In nur 20 Ländern bestand ein negativer Zusammenhang zwischen dem sozioökonomischen Status und dem wahrgenommenen Feedback von Schüler*innen. Im Distanzunterricht scheint es jedoch Unterschiede in der Feedbackhäufigkeit bezogen auf den sozioökonomischen Status von Schüler*innen zu geben. Wößmann et al. (2021) konnten in einer Elternbefragung zeigen, dass 10 % der Nichtakademiker-Kinder keine Rückmeldungen zu den bearbeiteten Aufgaben von ihren Lehrer*innen erhielten. Bei den Akademiker-Kindern waren es 6 %.

In einer aktuellen Studie von Schwab et al. (2022) wurde unter anderem der Zusammenhang zwischen Schüler*innenmerkmalen (sonderpädagogischer Förderbedarf, Geschlecht und Migrationshintergrund) und der Wahrnehmung von Feedback in Deutschland untersucht. Schüler*innen mit einem Migrationshintergrund nahmen das Feedback ihrer Lehrkräfte positiver wahr verglichen mit Schüler*innen ohne Migrationshintergrund. Auch bei Sortkær (2018) zeigte sich, dass Schüler*innen mit einem Migrationshintergrund häufiger Feedback erhalten. Eryilmaz und Sandoval-Hernández (2021) berichten von teilweise widersprüchlichen Befunden zwischen den Schüler*innen der ersten, zweiten Migrationsgeneration sowie Schüler*innen ohne Migrationshintergrund.

### Akademisches Selbstkonzept

Akademische Selbstkonzepte als „kognitive Repräsentation der eigenen spezifischen Fähigkeiten“ (Zurbriggen 2016, S. 15) hängen reziprok mit schulischen Leistungen und Leistungsmotivationen zusammen (Hansford und Hattie 1982; Marsh et al. 2005) und sind daher wichtige Bildungsoutcomes insbesondere für schwächere Schüler*innen (Weber und Freund 2017). Ein häufig rezipiertes Modell zum Selbstkonzept modellierten Shavelson et al. (1976), welche zwischen akademischen und nicht-akademischen Selbstkonzeptfacetten (sozial, emotional und physisch) unterscheiden. Im schulischen Kontext sind insbesondere akademische Selbstkonzepte von Interesse, da sie im Rahmen der Motivationsforschung ein bedeutungsvolles Schüler*innenmerkmal darstellen, welches enge Zusammenhänge zu schulischen Lern- und Leistungsprozessen von Schüler*innen aufweist (Shajek et al. 2006). Das akademische Selbstkonzept enthält generalisierte und selbstbezogene Fähigkeitskognitionen, „die sich auf die erbrachten Leistungen in den verschiedenen Schulfächern beziehen“ (Zeinz 2006, S. 1). Die das Selbstkonzept zum Teil konstituierenden sozialen Umfelder ergeben sich dabei unter anderem durch die Schule und Schulformzugehörigkeit an sich (Zurbriggen 2016). In einer Studie von Baudson et al. (2016) zeigte sich, in Bezug auf Unterschiede zwischen den Schulformen, dass Schüler*innen an Förder- und Hauptschulen über ein höheres akademisches Selbstkonzept verfügten im Vergleich zu Schüler*innen an Gesamt- und Realschulen sowie an Gymnasien. Zwischen den anderen Schulformen zeigten sich keine Unterschiede. Dieser Effekt lässt sich vermutlich auf die Genese von akademischen Selbstkonzepten durch unterschiedliche Referenzrahmen erklären (Baudson et al. 2016). Das entspricht auch den Annahmen von Marsh et al. (2006), nach welchen das Selbstkonzept als formbares Persönlichkeitsmerkmal gilt und stark von Kontext, Umfeld und Lebensereignissen beeinflusst wird. Faber et al. (2011) wiesen nach, dass Kinder mit Migrationshintergrund trotz gleicher bzw. schwächerer Leistungen keine schlechteren Selbstkonzepte als deutschsprachige Kinder aufweisen. Marsh und Parker (1984) fassen zusammen, dass der Zusammenhang zwischen dem akademischen Selbstkonzept und dem sozioökonomischen Status „has generally been found tovary between near zero and low positive“ (S. 215). In einer neueren Studie, die an chinesischen Schulen durchgeführt wurde, findet sich jedoch ein positiver Zusammenhang zwischen dem sozioökonomischen Status und dem akademischen Selbstkonzept von Schüler*innen (Li et al. 2020).

### Zusammenhang zwischen Feedback und akademischem Selbstkonzept

Der Zusammenhang von wahrgenommenem Feedback, dem akademischen Selbstkonzept sowie der sozialen Herkunft von Schüler*innen kann anhand des Erwartungs-Wert-Modells von Eccles (2007) verdeutlicht werden. Demnach wird die Wahrnehmung des Verhaltens der Lehrer*innen (z. B. Feedbackverhalten) durch die Merkmale der Schüler*innen (z. B. das Geschlecht) und dem kulturellen Milieu (z. B. die soziale Herkunft) der Schüler*innen bedingt. Diese Wahrnehmungen haben, neben den Schüler*innenmerkmalen, Auswirkungen auf die Ziele und Selbstschemata (u. a. das akademische Selbstkonzept) von Schüler*innen. Das Selbstkonzept beeinflusst sowohl den beigemessenen Wert einer Aufgabe, als auch die Erfolgserwartungen, welche sich wiederum beide auf die Leistungen auswirken (Eccles 2007).

Direkte und indirekte Rückmeldungen von Lehrkräften gelten daher als wichtige Faktoren für die Selbstkonzeptgenese von Schüler*innen (Chen et al. 2011; Blöte 1995) und der Zusammenhang zwischen Feedback und dem akademischen Selbstkonzept konnte mehrfach empirisch belegt werden (siehe Hoya 2019). Chen et al. (2011) zeigten beispielsweise, dass ein negativeres Feedback mit einem niedrigeren generellen Selbstkonzept der Schüler*innen zusammenhing, wohingegen positives akademisches Feedback ein positiver Prädiktor für ein positives akademisches Selbstkonzept war (siehe hierzu auch Blöte 1995). Insgesamt konnte in der Studie von Chen et al. (2011) belegt werden, dass Schüler*innen, die mehr positives akademisches Feedback erhielten, ein höheres generelles, akademisches und nicht-akademisches Selbstkonzept aufwiesen. Gleichwohl merkt Hoya (2019) an, dass die Forschungsergebnisse zum Zusammenhang von Feedback und dem Selbstkonzept teilweise widersprüchlich sind. Grund für die Inkonsistenz der Studien scheint die fehlende Berücksichtigung der Komplexität des Feedbackprozesses zu sein. Der Einfluss und das Zusammenwirken von unterschiedlichen Faktoren im Feedbackprozess wurde von mehreren Autor*innen angemerkt (Strijbos et al. 2021; Hattie und Timperley 2007; Narciss 2006; Kluger und DeNisi 1996). So stellt sich die Frage, ob – neben dem positivem und negativem wahrgenommen Feedback – weitere Merkmale von Feedback das akademische Selbstkonzept von Schüler*innen vorhersagen können. Sehen Schüler*innen z. B. einen Nutzen in dem Feedback von Lehrer*innen, hat dies einen positiven Effekt auf die Veränderung von Schulleistung (Harks et al. 2014). Ob die Wahrnehmung digitalen Feedbacks in Form von *Fairness, Akzeptanz* und *Nutzen* während der COVID-19 Pandemie einen Prädiktor für das akademische Selbstkonzept von Schüler*innen mit unterschiedlichem sozioökonomischem Hintergrund darstellt, wurde bislang nicht untersucht.

## Forschungsfragen und Hypothesen

In der vorliegenden Studie wird untersucht, wie häufig Schüler*innen digitales Feedback von ihren Lehrkräften während der COVID-19 Pandemie erhalten und wie sie das Feedback wahrnehmen. Dabei wird analysiert, welche Rolle die Schulform und die soziale Herkunft (sozioökonomischer Status und Migrationshintergrund) bei der Wahrnehmung von Feedback und dem akademischen Selbstkonzept spielen. Zudem wird überprüft, ob die *Feedbackhäufigkeit*, das wahrgenommene Feedback in Form von *Fairness, Nutzen* und *Akzeptanz* Prädiktoren für das akademische Selbstkonzept von Schüler*innen darstellen. Folgende Forschungsfragen werden im Rahmen der vorliegenden Studie beantwortet:

### 1.

Wie oft erhalten Schüler*innen Feedback von ihren Lehrkräften im Distanzlernen und welche Unterschiede zeigen sich in Abhängigkeit von der sozialen Herkunft und der Schulform?

Auf Grundlage bisheriger Befunden zur wahrgenommenen Feedbackhäufigkeit von Schüler*innen mit Migrationshintergrund (Schwab et al. 2022; Sortkær 2018) gehen wir davon aus, dass Schüler*innen mit einem Migrationshintergrund häufiger Feedback erhalten, als Schüler*innen ohne einen Migrationshintergrund (**H1**).

Basierend auf den Forschungsergebnissen zur wahrgenommenen Feedbackhäufigkeit von Eltern im Distanzlernen und dem sozioökonomischen Status (Wößmann et al. 2021) nehmen wir an, dass Schüler*innen mit einem hohen sozioökonomischen Status häufiger Feedback erhalten (**H2**).

Laut Helm et al. (2021) gibt es während der COVID-19 Pandemie keine Unterschiede in der wahrgenommenen Feedbackhäufigkeit nach Schulform. Die Ergebnisse stammen jedoch aus Österreich oder beruhen auf Angaben von Eltern. Da im gegliederten Schulsystem in Deutschland schulformspezifische Unterschiede in der Zusammensetzung der Schüler*innenschaft und Institutionelle Unterschiede bestehen (Baumert et al. 2006), vermuten wir, dass die wahrgenommene Feedbackhäufigkeit von Schüler*innen sich nach der Schulform unterscheidet (**H3**).

### 2.

Welche Unterschiede zeigen sich bei der wahrgenommenen *Fairness*, dem *Nutzen* und der *Akzeptanz* des digitalen Feedbacks sowie beim akademischen Selbstkonzept zwischen verschiedenen Gruppen (sozioökonomischer Status, Migrationshintergrund und Schulform)?

Einhergehend mit Forschungsbefunden zum Zusammenhang vom sozioökonomischen Status und dem wahrgenommenen Feedback von Schüler*innen (Eryilmaz und Sandoval-Hernández 2021; Sortkær 2019) gehen wir von schwachen Unterschieden in der Wahrnehmung des Feedbacks zwischen Schüler*innen mit einem hohen sozioökonomischen Status und Schüler*innen mit einem niedrigen sozioökonomischen Status aus (**H4**).

Basierend auf vorliegenden Studien (Schwab et al. 2022; Eryilmaz und Sandoval-Hernández 2021; Sortkær 2018) vermuten wir, dass es Unterschiede in der Wahrnehmung von Feedback zwischen den Schüler*innen der ersten und zweiten Migrationsgeneration sowie Schüler*innen ohne Migrationshintergrund gibt (**H5**).

Unterschiede in der Wahrnehmung von Feedback von Schüler*innen nach der Schulform wurden bislang nicht untersucht. Da die kontextuellen Bedingungen des Feedbacks bei der Verarbeitung von Feedback eine Rolle spielen (Strijbos und Müller 2014), gehen wir von der Annahme aus, dass es Unterschiede in der Wahrnehmung des Feedbacks nach der Schulform gibt (**H6**).

Auf der Grundlage von bisherigen Befunden zur sozialen Herkunft und dem akademischen Selbstkonzept (Li et al. 2020; Faber et al. 2011; Marsh und Parker 1984) nehmen wir an, dass es keine Unterschiede in der Ausprägung des akademischen Selbstkonzeptes nach dem Migrationshintergrund gibt. Darüber hinaus gehen wir davon aus, dass Schüler*innen mit einem hohen sozioökonomischen Status ein höheres akademisches Selbstkonzept haben, als Schüler*innen mit einem niedrigen sozioökonomischen Status. In Anlehnung an Baudson et al. (2016) nehmen wir an, dass sich keine Unterschiede im akademischen Selbstkonzept nach der Schulform zeigen, da in unserer Stichprobe keine Förder- und Hauptschüler*innen vertreten sind (**H7**).

### 3.

Können das wahrgenommene Feedback, die Feedbackhäufigkeit und die soziale Herkunft das akademische Selbstkonzept der Schüler*innen im Distanzlernen vorhersagen?

Einhergehend mit bisherigen Befunden zum Zusammenhang von Feedback und dem akademischen Selbstkonzept (Chen et al. 2011; Blöte 1995) gehen wir von der Annahme aus, dass das wahrgenommene Feedback sowie die Feedbackhäufigkeit positive Prädiktoren für das akademische Selbstkonzept darstellen (**H8**).

Beruhend auf Forschungsergebnissen zur sozialen Herkunft und dem akademischen Selbstkonzept von Schüler*innen (Baudson et al. 2016; Li et al. 2020; Faber et al. 2011; Marsh und Parker 1984), vermuten wir, dass der sozioökonomische Status das akademische Selbstkonzept vorhersagen kann (**H9**).

## Methode

### Stichprobe und Durchführung

In einer bundesweiten Online-Umfrage im Zeitraum von Mai bis September 2020 wurden insgesamt 668 Schüler*innen im Alter von 11 bis 20 Jahren (*M* = 16,04 Jahre, *SD* = 1,88) befragt. Die Schüler*innen der Umfrage kamen aus Hamburg (30 %), Niedersachsen (28 %), Nordrhein-Westfahlen (17 %), Bayern (6 %), Baden-Württemberg (4 %), Schleswig-Holstein (3 %), Hessen (3 %), Rheinland-Pfalz (2 %), Berlin (2 %), Sachsen (1 %), Brandenburg, Bremen, Mecklenburg-Vorpommern, Saarland und Sachsenanhalt (zusammen insgesamt 3 %). Der Fragebogen wurde per Email an Schulen und Lehrkräfte geschickt sowie über die sozialen Medien (u. a. Twitter und Instagram) verbreitet. Vor der Datenerhebung wurde eine digitale Einverständniserklärung der Erziehungsberechtigten eingefordert. Zusätzlich erhielten alle Schüler*innen einen Überblick über die Datenerhebung, die Anonymität ihrer Antworten und es wurde ihnen die Möglichkeit gegeben, jederzeit die Befragung abzubrechen. Die Beantwortung des Fragebogens dauerte zwischen 10 und 20 min.

Stadtteilschulen, Oberschulen und Gemeinschaftsschulen wurden unter dem Begriff Sekundarschulen mit Oberstufe zusammengefasst. Je nach Bundesland ersetzten integrierte Sekundarstufen mit eigener Oberstufe die Real‑, Haupt- und Gesamtschulen (Hurrelmann 2013). Trotzdem gibt es weiterhin in Deutschland Gesamtschulen und Realschulen. Deshalb wurden diese Schulformen in der Verteilung der Gesamtstichprobe (siehe Tab. 1) separat aufgeführt.

**Tab. 1:** Verteilung der Gesamtstichprobe (*N* = 668) nach Schulform, Klassenstufe, Geschlecht, Migrationshintergrund und sozioökonomischem Status

	Gesamt	Sekundarschulen mit Oberstufe	Gesamtschulen	Gymnasien	Realschulen
Anzahl Schüler*innen	668	182	92	333	61
*Klassenstufen (in %)*					
5. bis 8. Klasse	149 (*22,3* *%*)	65 (*35,7* *%*)	15 (*16,3* *%*)	52 (*15,6* *%*)	17 (*27,9* *%*)
9. bis 10. Klasse	302 (*45,2* *%*)	83 (*45,6* *%*)	33 (*35,9* *%*)	142 (*42,6* *%*)	44 (*72,1* *%*)
11. bis 13. Klasse	217 (*32,5* *%*)	34 (*18,7* *%*)	44 (*47,8* *%*)	139 (*41,7* *%*)	–
*Geschlecht (in %)*					
Weiblich	501 (*75* *%*)	128 (*70,3* *%*)	72 (*78,3* *%*)	245 (*73,6* *%*)	56 (*91,8* *%*)
Männlich	167 (*25* *%*)	54 (*29,7* *%*)	20 (*21,7* *%*)	88 (*26,4* *%*)	5 (*8,2* *%*)
*Migrationshintergrund (in %)*					
Erste Migrationsgeneration	43 (*6,4* *%*)	12 (*6,6* *%*)	5 (*5,4* *%*)	20 (*6* *%*)	6 (*9,8* *%*)
Zweite Migrationsgeneration	233 (*34,9* *%*)	59 (*32,4* *%*)	32 (*34,8* *%*)	111 (*33,3* *%*)	31 (*50,8* *%*)
Kein Migrationshintergrund	392 (*58,7* *%*)	111 (*61* *%*)	55 (*59,8* *%*)	202 (*60,7* *%*)	24 (*39,3* *%*)
*HISEI eingeteilt in Quartile (in %)*					
0–25 % Quartil	168 (*25,1* *%*)	53 (*29,1* *%*)	30 (*32,6* *%*)	60 (*18* *%*)	25 (*41* *%*)
25–50 % Quartil	179 (*26,8* *%*)	44 (*24,2* *%*)	23 (*25* *%*)	94 (*28,2* *%*)	18 (*29,5* *%*)
50–75 % Quartil	154 (*23,1* *%*)	54 (*29,7* *%*)	19 (*20,7* *%*)	69 (*20,7* *%*)	12 (*19,7* *%*)
75–100 % Quartil	167 (*25* *%*)	31 (*17* *%*)	20 (*21,7* *%*)	110 (*33* *%*)	6 (*9,8* *%*)

Es wurde bei der Zuordnung der Schüler*innen der ersten Migrationsgeneration (*M*_HISEI_ = 61,83; *SD* = 20,85), der zweiten Migrationsgeneration (*M*_HISEI_ = 53,97; *SD* = 20,01) und bei Schüler*innen ohne Migrationshintergrund (*M*_HISEI_ = 61,63; *SD* = 18,72) auf eine weitere Differenzierung zwischen EU und Nicht-EU-Bürgern verzichtet

*HISEI* höchster ISEI (International Socio-economic Index of Occupational Status), *Stadtteilschulen* Gemeinschaftsschulen, Oberschulen, *erste Migrationsgeneration* Schüler*in ist im Ausland geboren, *zweite Migrationsgeneration* ein Elternteil oder beide Elternteile sind im Ausland geboren

### Eingesetzte Skalen

Die demographischen Angaben (Geschlecht, Schulform, Alter, Migrationshintergrund) wurden im Rahmen der Online-Umfrage erfasst. In Anlehnung an Stanat und Segeritz (2009) wurde zwischen der ersten und zweiten Migrationsgeneration unterschieden. Schüler*innen der ersten Migrationsgeneration sind selbst zugewandert. Zur zweiten Migrationsgeneration gehören Schüler*innen, die in Deutschland geboren sind, aber von denen mindestens ein Elternteil im Ausland geboren ist.

#### Sozioökonomischer Status

Zur Messung des sozioökonomischen Status wurde der International Socio-Economic Index of occupational status (ISEI) nach Ganzeboom et al. (1992) genutzt. Hierfür wurden die Schüler*innen nach den Berufen ihrer Eltern befragt. Der ISEI basiert auf dem International Standard Classification of Occupation (ISCO) und bietet die Möglichkeit, die Einkommens- und Bildungsunterschiede zwischen Berufskategorien abzubilden. Die Werte reichen von 16 Punkten (ungelernte Hilfskräfte) bis 90 Punkten (Richter*in). Diese wurden anhand der International Standard Classification of Occupations 2008 (ISCO-08) kodiert und in den ISEI umkodiert. Der sozioökonomische Status einer Familie wird über den höchsten ISEI (HISEI) abgebildet. Dafür werden die Werte des ISEIs von beiden Elternteile verglichen und der höchste Wert (HISEI) wird ausgewählt.

#### Wahrgenommenes Feedback und Feedbackhäufigkeit

Um das *wahrgenommene Feedback* der Schüler*innen zu erfassen, wurde der Feedback Perceptions Questionnaire (FPQ) von Strijbos et al. (2010) mit den Subskalen *Fairness* (Beispielitem: Ich bin zufrieden mit dem Feedback; α = 0,88); *Nutzen* (Beispielitem: Ich finde das Feedback nützlich; α = 0,90) und *Akzeptanz* (Beispielitem: Ich bin mit dem Feedback einverstanden; α = 0,72) genutzt. Der FPQ wurde in einer aktuellen Studie von Strijbos et al. (2021) umfassend validiert und Messinvarianz in Bezug auf Geschlecht, Schulform und Klassenstufe wurde sichergestellt. Zusätzlich wurden die Schüler*innen befragt, wie häufig sie Feedback von ihren Lehrkräften erhalten („weniger als 1 × pro Woche, 1 × pro Woche, alle zwei Tage, jeden Tag, mehrmals täglich“).

#### Akademisches Selbstkonzept

Zur Erfassung des akademischen Selbstkonzepts wurde die Subskala *Generelles schulisches Selbstkonzept* aus dem *Fragebogen zur Erfassung multipler Selbstkonzeptfacetten bei Förderschülern (Schwerpunkt Lernen) und Regelschülern *(FSKFR) von Weber und Freund (2017) eingesetzt (α = 0,86). Die Items wurden auf einer 4‑stufigen Ratingskala beantwortet (1 = Nein bis 4 = Ja). Beispielitems sind „Die meisten Aufgaben in der Schule fallen mir leicht“ oder „In meiner Klasse gehöre ich zu den guten Schüler*innen“. Die Items beinhalten somit auch die soziale Bezugsnorm (Weber und Freund 2017). Der FSKFR wurde umfassend validiert (Weber und Freund 2017, 2017a) und die Messinvarianz in Bezug auf Geschlecht und Schulform wurde sichergestellt.

### Statistische Analysen

Die erhobenen Daten wurden mit der Statistik- und Analysesoftware IBM SPSS (Statistics 28) aufbereitet und analysiert. Die Imputation fehlender Werte wurde über den Expectation Maximization (EM)-Algorithmus vorgenommen (Leonhart 2017). Zur Beantwortung der ersten beiden Forschungsfragen wurden einfaktorielle ANOVAs berechnet mit Bonferroni korrigierten Post-hoc Tests. Bei der dritten Fragestellung wurde eine multiple Regressionsanalyse berechnet mit dem akademischen Selbstkonzept als abhängige Variable und Geschlecht, Klassenstufe, Schulform, Migrationshintergrund, sozioökonomischer Status, Feedbackhäufigkeit sowie *Fairness, Nutzen* und *Akzeptanz* des Feedbacks als unabhängige Variablen.

## Ergebnisse

### Feedbackhäufigkeit

Bei der wahrgenommenen *Feedbackhäufigkeit* im Distanzlernen konnten Unterschiede in Abhängigkeit von der sozialen Herkunft und der Schulform festgestellt werden (siehe Tab. 2). Die Ergebnisse einer ANOVA zur *Feedbackhäufigkeit *bestätigen **H1** partiell und zeigten, dass Schüler*innen der ersten Migrationsgeneration signifikant (*p* < 0,001) mehr Feedback von ihren Lehrkräften wahrnahmen, als Schüler*innen der zweiten Migrationsgeneration und Schüler*innen ohne Migrationshintergrund.

**Tab. 2:** Wahrgenommene *Häufigkeit, Fairness, Nutzen* und *Akzeptanz* des digitalen Feedbacks sowie *akademisches Selbstkonzept*

	Wahrgenommenes Feedback									
	Häufigkeit		Fairness		Nutzen		Akzeptanz		ASK	
	M	SD	M	SD	M	SD	M	SD	M	SD
*Gesamt*	1,99	0,91	2,70	0,98	2,54	1,02	2,69	0,86	2,65	0,85
*Geschlecht*										
Weiblich	1,96	0,91	**2,74**	**0,98**	**2,60**	**1,02**	2,71	0,88	**2,61**	**0,87**
Männlich	2,06	0,93	**2,55**	**0,96**	**2,35**	**1,00**	2,61	0,81	**2,76**	**0,85**
*Klassenstufen*										
5. bis 8. Klasse	**2,42**	**1,05**	**2,96**	**1,00**	2,67	1,07	**2,93**	**0,84**	2,59	0,88
9. bis 10. Klasse	**1,95**	**0,87**	**2,71**	**0,99**	2,54	1,04	**2,71**	**0,85**	2,68	0,88
11. bis 13. Klasse	**1,74**	**0,75**	**2,50**	**0,91**	2,46	0,95	**2,49**	**0,86**	2,66	0,79
*Schulform*										
Sekundarschulen mit Oberstufe	**2,18**	**0,93**	**2,93**	**0,93**	**2,75**	**1,00**	**2,85**	**0,78**	2,63	0,83
Gesamtschulen	**1,87**	**0,86**	2,78	0,94	2,48	0,94	**2,90**	**0,85**	2,59	0,88
Gymnasien	**1,92**	**0,89**	**2,53**	**0,97**	**2,44**	**1,03**	**2,50**	**0,88**	2,68	0,85
Realschulen	1,99	0,96	2,76	1,12	2,59	1,06	**2,87**	**0,83**	2,59	0,93
*Migrationshintergrund*										
Erste Migrationsgeneration	**2,64**	**1,22**	2,78	1,07	2,69	1,08	**2,39**	**0,91**	2,75	0,82
Zweite Migrationsgeneration	**1,94**	**0,85**	2,77	0,99	2,52	0,99	**2,77**	**0,81**	2,56	0,87
Kein Migrationshintergrund	**1,95**	**0,88**	2,64	0,96	2,54	1,03	2,67	0,88	2,69	0,85
*HISEI*										
0–25 % Quartil	**2,10**	**0,86**	2,69	0,94	2,43	0,85	2,75	0,78	**2,46**	**0,76**
25–50 % Quartil	**2,09**	**0,97**	2,68	0,98	2,50	1,09	2,71	0,86	**2,55**	**0,88**
50–75 % Quartil	**1,93**	**0,89**	2,67	1,03	2,54	1,07	2,65	0,90	**2,77**	**0,85**
75–100 % Quartil	**1,83**	**0,90**	2,73	0,99	2,69	1,03	2,63	0,92	**2,87**	**0,87**

Fett markiert bei signifikanten Unterschieden zu mindestens einer anderen Gruppe der gleichen Kategorie

*HISEI* höchster ISEI (International Socio-economic Index of Occupational Status), *Sekundarschulen mit Oberstufe* Stadtteilschulen, Gemeinschaftsschulen, Oberschulen, *erste Migrationsgeneration* Schüler*in ist im Ausland geboren, *zweite Migrationsgeneration* ein Elternteil oder beide Elternteile sind im Ausland geboren, *ASK* Akademisches Selbstkonzept

Unterschiede nach dem sozioökonomischen Status (HISEI) der Schüler*innen fanden sich ebenfalls für die wahrgenommene *Feedbackhäufigkeit*. Schüler*innen mit einem niedrigen sozioökonomischen Status (0–25 % Quartil) erhielten erwartungswidrig am häufigsten Feedback (**H2**). Sie unterschieden sich signifikant (*p* < 0,05) von Schüler*innen mit einem hohen sozioökonomischen Status (50–75 HISEI-Quartil und 75–100 HISEI-Quartil). Schüler*innen des 25–50 HISEI-Quartils nahmen signifikant häufiger Feedback wahr, als Schüler*innen des 75–100 HISEI-Quartils.

Im Hinblick auf die Schulform nahmen Schüler*innen der Sekundarschulen mit Oberstufe signifikant (*p* < 0,05) häufiger Feedback wahr, als Schüler*innen der Gymnasien und Gesamtschulen (**H3**).

### Wahrgenommenes Feedback sowie akademisches Selbstkonzept

Es zeigten sich wider Erwarten keine Unterschiede bei der Wahrnehmung des Feedbacks nach dem sozioökonomischen Status der Schüler*innen (**H4**).

Eine ANOVA zeigte erwartungsgemäß signifikante (*p* < 0,05) Unterschiede nach dem Migrationshintergrund (**H5**). Schüler*innen der zweiten Migrationsgeneration akzeptierten das Feedback von ihren Lehrer*innen eher, als Schüler*innen der ersten Migrationsgeneration (siehe Tab. 2).

Unsere Annahmen bestätigend zeigten sich Unterschiede in der Wahrnehmung des Feedbacks nach der Schulform (**H6**). Die *Fairness*, der *Nutzen* und die *Akzeptanz* des Feedbacks wurde von Gymnasialschüler*innen signifikant (*p* < 0,01) schlechter wahrgenommen, als von Schüler*innen der Sekundarschulen mit Oberstufe (siehe Tab. 2). Darüber hinaus hatten Schüler*innen der Gesamtschulen eine signifikant (*p* < 0,001) höhere *Akzeptanz* des Feedbacks, als Schüler*innen der Gymnasien. Auch Realschüler*innen unterschieden sich signifikant (*p* < 0,05) von Gymnasiasten hinsichtlich der *Akzeptanz* des wahrgenommenen Feedbacks.

Es wurde eine einfaktorielle ANOVA berechnet, um zu untersuchen, ob es einen Unterschied hinsichtlich des *akademischen Selbstkonzeptes* in Abhängigkeit von der sozialen Herkunft und der Schulform gab. Erwartungsgemäß zeigten sich in dem akademischen Selbstkonzept ausschließlich Unterschiede nach dem sozioökonomischen Status der Schüler*innen (**H7**). Schüler*innen mit einem hohen sozioökonomischen Status (75–100 HISEI-Quartil) berichteten ein signifikant (*p* < 0,001) höheres *akademisches Selbstkonzept,* als Schüler*innen mit einem niedrigen sozioökonomischen Status (0–25 HISEI-Quartil). Darüber hinaus unterschieden sich Schüler*innen mit einem hohen sozioökonomischen Status signifikant (*p* < 0,01) von Schüler*innen des 25–50 HISEI-Quartils. Auch Schüler*innen des 50–75 HISEI-Quartils zeigten ein signifikant (*p* < 0,01) höheres *akademisches Selbstkonzept*, verglichen mit Schüler*innen mit einem niedrigen sozioökonomischen Status.

Es zeigten sich keine schulformbezogenen Unterschiede und Unterschiede nach dem Migrationshintergrund in dem *akademischen Selbstkonzept*.

### Zusammenhang zwischen Feedback und akademischem Selbstkonzept

Zur Vorhersage des akademischen Selbstkonzepts im Distanzunterricht wurde eine multiple Regressionsanalyse durchgeführt (siehe Tab. 3). Dabei wurden die soziale Herkunft, die Schulform, das Geschlecht, die Klassenstufe und das wahrgenommene Feedback als Prädiktoren einbezogen. Die Prädiktorvariablen konnten zusammen 21 % der Varianz des akademischen Selbstkonzeptes erklären. *Fairness* (*β* = 0,28) und *Nutzen* (*β* = 0,22) des wahrgenommenen Feedbacks, HISEI (*β* = 0,10) und Geschlecht (*β* = 0,12) der Schüler*innen sowie die Schulform (*β* = 0,07) waren erwartungsgemäß signifikante Prädiktoren für das akademische Selbstkonzept im Distanzlernen (H8 und H9).

**Tab. 3:** Regressionsanalyse zur Vorhersage des akademischen Selbstkonzeptes

	Akademisches Selbstkonzept			
	B	SE	β	*p*
Geschlecht	**0,239**	**0,069**	**0,121**	**<** **0,001**
Klassenstufe	0,027	0,017	0,058	0,114
Schulform	**0,064**	**0,031**	**0,074**	**<** **0,05**
MH	− 0,051	0,049	− 0,037	0,295
HISEI	**0,006**	**0,002**	**0,136**	**<** **0,001**
Feedbackhäufigkeit	0,032	0,035	0,034	0,360
Fairness des Feedbacks	**0,241**	**0,048**	**0,277**	**<** **0,001**
Nutzen des Feedbacks	**0,188**	**0,038**	**0,224**	**<** **0,001**
Akzeptanz des Feedbacks	− 0,050	0,046	− 0,050	0,280
*R*^2^	0,21			

Geschlecht (1 = weiblich, 2 = männlich)

*MH* Migrationshintergrund, *HISEI* höchster ISEI (International Socio-economic Index of Occupational Status)

## Diskussion

Ziel der Studie war es, zu untersuchen, welche Rolle die soziale Herkunft (sozioökonomischer Status und der Migrationshintergrund) und die Schulform bei der *Wahrnehmung der Häufigkeit, der Wahrnehmung der Fairness, des Nutzens und der Akzeptanz von digitalem Feedback* und dem *akademischen Selbstkonzept* während der COVID-19 Pandemie spielt. In einem zweiten Schritt wurde untersucht, inwiefern die soziale Herkunft, die Schulform, die Feedbackhäufigkeit und das wahrgenommene Feedback der Schüler*innen das *akademische Selbstkonzept* vorhersagen können.

Entsprechend unseren Annahmen gaben Schüler*innen der ersten Migrationsgeneration an, dass sie häufiger Feedback von ihren Lehrkräften erhielten als Schüler*innen der zweiten Migrationsgeneration, oder Schüler*innen ohne Migrationshintergrund (**H1**). Auch Schwab et al. (2022) sowie Sortkær (2018) kamen in ihren Studien zu einem ähnlichen Ergebnis. Dies mag damit zusammenhängen, dass den Lehrkräften die sprachlichen Schwierigkeiten der Schüler*innen bewusst sind (Sortkær 2018) und sie deshalb mehr Feedback geben. Der sozioökonomische Status von Schüler*innen der ersten Migrationsgeneration entsprach dem sozioökonomischen Status von Schüler*innen ohne Migrationshintergrund. Eine mögliche Erklärung hierfür ist, dass Schüler*innen der ersten Migrationsgeneration aus anderen EU-Ländern stammen (Schiefer 2013). Jedoch erhielten Schüler*innen mit einem hohen sozioökonomischen Status am wenigsten Feedback. Diese widersprüchlichen Ergebnisse könnten durch die hohe Standardabweichung in der *wahrgenommenen Feedbackhäufigkeit* bei Schüler*innen der ersten Migrationsgeneration erklärt werden.

Bemerkenswert sind unsere Ergebnisse zur *wahrgenommenen Feedbackhäufigkeit* in Abhängigkeit vom sozioökonomischen Status der Schüler*innen. Entgegen unserer Annahme konnten in unserer Studie nicht bestätigt werden, dass Schüler*innen mit einem hohen sozioökonomischen Status häufiger Feedback erhalten (**H2**). Diese Ergebnisse widersprechen den Befunden einer Elternbefragung während der COVID-19 Pandemie (Wößmann et al. 2021). Möglicherweise hängt die Differenz mit den unterschiedlichen Formulierungen der Fragen zur *wahrgenommen Feedbackhäufigkeit* zusammen. Während bei Wößmann et al. (2021) nach Rückmeldungen zu bearbeiteten Aufgaben gefragt wurden, haben wir nach der Häufigkeit von Feedback im Allgemeinen gefragt. Die Diskrepanz der *wahrgenommenen Feedbackhäufigkeit* könnte auch an den gruppenspezifischen Wahrnehmungen liegen. So nehmen bei Blöte (1995) Schüler*innen und Lehrer*innen positives und negatives Feedback unterschiedlich wahr.

Die Hypothese, dass die wahrgenommene Feedbackhäufigkeit von Schüler*innen sich nach der Schulform unterscheidet, konnte bestätigt werden (**H3**). Schüler*innen der Sekundarschulen mit Oberstufe nahmen häufiger Feedback wahr, als Schüler*innen der Gymnasien und Gesamtschulen. Die Ergebnisse deuten auf institutionelle Unterschiede und Unterschiede in der Zusammensetzung der Schüler*innenschaft hin (Baumert et al. 2006).

Insgesamt weisen unsere Ergebnisse zur *wahrgenommenen Feedbackhäufigkeit *nach der sozialen Herkunft darauf hin, dass Lehrkräfte aller Schulformen möglicherweise die Risikogruppen des deutschen Bildungssystems (Weis et al. 2018; Mahler und Kölm 2018; Henschel et al. 2018; Stubbe et al. 2019) kennen und diesen häufiger Rückmeldungen geben.

Unsere vierte Hypothese konnte nicht bestätigt werden – es zeigten sich keine Unterschiede bei der *Wahrnehmung des Feedbacks* im Hinblick auf den sozioökonomischen Status der Schüler*innen (**H4**). Eryilmaz und Sandoval-Hernández (2021) weisen darauf hin, dass der sozioökonomische Status nur eine kleine Rolle bei der *Wahrnehmung von Feedback* spielt. Unsere Befunde unterstreichen diese Erkenntnis. Zusätzlich konnten wir zeigen, dass es keine Unterschiede bei der *Fairness*, dem *Nutzen* und der *Akzeptanz* des *wahrgenommenen Feedbacks* im Distanzlernen in Bezug auf den sozioökonomischen Status gab. Diese Ergebnisse sollten auch im Präsenzunterricht überprüft werden.

Die Annahme, dass sich Unterschiede in der Wahrnehmung von Feedback nach dem Migrationshintergrund zeigen, konnte nur teilweise bestätigt werden (**H5**). In unserer Studie konnten keine Unterschiede zwischen Schüler*innen ohne Migrationshintergrund und Schüler*innen mit einem Migrationshintergrund gefunden werden. Dieses Ergebnis widerspricht den Befunden von Schwab et al. (2022). Jedoch *akzeptierten* Schüler*innen der zweiten Migrationsgeneration das Feedback von ihren Lehrkräften eher, als Schüler*innen der ersten Migrationsgeneration, obwohl Schüler*innen der ersten Migrationsgeneration am häufigsten Feedback erhielten. Die Feedbackquelle (Lehrkraft) wird als einer der wichtigsten Faktoren bei der *Akzeptanz* des *wahrgenommen Feedbacks* angesehen (Strijbos et al. 2010). Warum Schüler*innen der ersten Migrationsgenration das Feedback der Lehrkräfte weniger akzeptierten, müsste in Studien zur Gestaltung des gesendeten Feedbacks untersucht werden. Trotzdem ergänzen unsere Befunde die Ergebnisse von Schwab et al. (2022), indem wir zeigen konnten, wie Schüler*innen mit einem Migrationshintergrund das Feedback ihrer Lehrkräfte wahrnehmen.

Unsere Hypothese, dass es Unterschiede in der Wahrnehmung von Feedback nach der Schulform gibt, konnte bestätigt werden (**H6**). Gymnasialschüler*innen nahmen die *Fairness*, den *Nutzen *und die *Akzeptanz* des Feedbacks im Distanzlernen schlechter wahr, als Schüler*innen der Sekundarschulen mit Oberstufe. Bei der *Akzeptanz* des Feedbacks unterschied sich das Gymnasium darüber hinaus auch von Realschulen und Gesamtschulen. Die Unterschiede in der Wahrnehmung von Feedback zwischen den Schulformen dürften unter anderem darauf zurückzuführen sein, dass Schüler*innen an Gymnasien das Feedback differenzierter wahrnahmen. Grund hierfür ist, dass Schüler*innen mit einem hohen sozioökonomischen Status (am Gymnasium haben 33 % der Schüler*innen einen hohen HISEI) am ehesten Verständnis für die pädagogische Sprache des Feedbacks (Sortkær 2018) mitbringen. Neben den möglichen Kompositionseffekten könnten auch institutionelle Unterschiede eine Rolle bei der *Wahrnehmung von Feedback* spielen. In unserer Studie wurden am Gymnasium häufiger E‑Mails als Kommunikationsmittel eingesetzt, während an den anderen Schulformen häufiger eine Kommunikationssoftware oder das Telefon verwendet wurde.^1^ Eine Kommunikationssoftware ermöglicht der Lehrkraft, Feedback über eine Chatfunktion oder per Videocall zu geben. Auch Rückfragen der Schüler*innen werden über eine Kommunikationssoftware erleichtert. Wenn Feedback innerhalb einer Schulform stärker als Selektions- oder Entwicklungsinstrument (Strijbos und Müller 2014) eingesetzt wird, kann dies die *Wahrnehmung von Feedback* beeinflussen. Möglicherweise wird am Gymnasium Feedback stärker als Selektionsinstrument verwendet. Diese Interpretation müsste jedoch in weiteren Studien empirisch untermauert werden.

Die Annahme, dass Schüler*innen mit einem hohen sozioökonomischen Status ein höheres *akademisches Selbstkonzept* haben, als Schüler*innen mit einem niedrigen sozioökonomischen Status, konnte bestätigt werden (**H7**). Die Ergebnisse der vorliegenden Studie verdeutlichen zudem wie angenommen, dass sich keine Unterschiede in der Ausprägung des *akademischen Selbstkonzeptes* im Hinblick auf den Migrationshintergrund und die Schulform zeigen. Diese Ergebnisse stehen in Einklang mit Studien zum akademischen Selbstkonzept und der sozialen Herkunft von Schüler*innen (z. B. Li et al. 2020).

Unsere Annahme, dass das wahrgenommene Feedback sowie die Feedbackhäufigkeit positive Prädiktoren für das akademische Selbstkonzept darstellen, konnte teilweise bestätigt werden (**H8**). Mithilfe einer Regressionsanalyse konnten wir zeigen, dass die wahrgenommene *Fairness* und der *Nutzen* des Feedbacks positive Prädiktoren für das *akademische Selbstkonzept* im Distanzunterricht waren. Die *wahrgenommene Feedbackhäufigkeit*, als quantitatives Maß des Feedbacks, kann das akademische Selbstkonzept während der COVID-19 Pandemie nicht vorhersagen. Die Ergebnisse verdeutlichen die Wichtigkeit des *wahrgenommenen Feedbacks *bei der Genese des *akademischen Selbstkonzeptes* und zeigen gleichzeitig, dass nicht die *Häufigkeit des Feedbacks* entscheidend ist, sondern die wahrgenommene Qualität.

Unsere letzte Hypothese, dass der sozioökonomische Status das akademische Selbstkonzept im Distanzlernen vorhersagen kann, konnte bestätigt werden (**H9**). Dieses Ergebnis dürfte unter anderem darauf zurückzuführen sein, dass Schüler*innen mit einem hohen sozioökonomischen Status im Elternhaus ein kognitiv anregungsreicheres Umfeld angeboten bekommen, welches das akademische Selbstkonzept positiv beeinflusst.

Insgesamt bestätigt unser Modell die vorliegenden Forschungsbefunde zum Zusammenhang von Feedback und dem *akademischen Selbstkonzept* (u. a. Hoya 2019; Chen et al. 2011; Blöte 1995). Darüber hinaus konnten wir bisherige Forschungsbefunde erweitern, indem wir zeigen konnten, dass die wahrgenommene *Fairness* und der *Nutzen* des Feedbacks das *akademische Selbstkonzept* im Distanzlernen deutlich vorhersagen können.

### Limitationen

Limitationen der Studie betreffen vor allem die Art der Datenerhebung und das Forschungsdesign. Da unsere Studie digital durchgeführt wurde und über die sozialen Medien verbreitet wurde, ist unsere Stichprobe nicht repräsentativ und die Ergebnisse müssen daher mit Vorsicht interpretiert werden. Um eine große Reichweite zu generieren, haben wir unseren Fragebogen durch eine weibliche Influencerin auf Instagram verbreiten lassen. 65 % der Personen, die ihr auf Instagram folgen, sind weiblich, wodurch die Verteilung der Geschlechter beeinflusst wurde. Insbesondere Schüler*innen mit einer mangelhaften technischen Ausstattung sowie Schüler*innen ohne Deutschkenntnisse sind in unserer Studie nicht vertreten. Da es sich um Querschnittsdaten handelt, können keine kausalen Schlüsse gezogen werden. Insbesondere im Hinblick auf das akademische Selbstkonzept und das wahrgenommene Feedback wäre eine längsschnittlich angelegte Untersuchung interessant gewesen. Die Gestaltung des Feedbacks sowie die Feedbacknachricht spielen eine wesentliche Rolle bei der Verarbeitung von Feedback (Strijbos und Müller 2014). Des Weiteren können gleiche Feedback-Ereignisse von Schüler*innen und Lehrer*innen unterschiedlich wahrgenommen werden (z. B. Hoya 2019). Deshalb wäre es in zukünftiger Forschung wichtig, auch das gesendete Feedback der Lehrer*innen zu erheben.

### Implikationen für die schulische Praxis

Es konnte mit der vorliegenden empirischen Arbeit gezeigt werden, dass Unterschiede in der *Wahrnehmung von Feedback* besonders nach der Schulform bestehen. Diese hängen wahrscheinlich mit der Zusammensetzung der Schüler*innenschaft und institutionellen Unterschieden zusammen. So nehmen Schüler*innen am Gymnasium das Feedback der Lehrkraft als weniger *fair* wahr und sehen seltener einen *Nutzen* darin. Eine differenzierte Debatte über Feedback als Selektions- oder Entwicklungsinstrument könnte an Gymnasien ein Bewusstsein für das Potenzial von (digitalem) Feedback und damit einhergehend zu einer Verbesserung des akademischen Selbstkonzeptes führen.

Schüler*innen mit einem niedrigen sozioökonomischen Status und einem Migrationshintergrund erhalten am häufigsten Feedback und weisen kaum Unterschiede in der Wahrnehmung des Feedbacks auf. Dieses Ergebnis deutet darauf hin, dass Lehrer*innen auch im Distanzlernen sozial benachteiligte Schüler*innen erkennen und Feedback adaptiv gestalten. Dennoch besteht eine Differenz zwischen der Feedbackhäufigkeit und der *wahrgenommenen Akzeptanz* des *Feedbacks *bei Schüler*innen der ersten Migrationsgeneration. Obwohl sie am häufigsten Feedback erhalten, sind sie nicht mit dem Feedback einverstanden und lehnen es häufiger ab, als Schüler*innen der zweiten Migrationsgeneration. Die Reflexion über die fehlende Akzeptanz des Feedbacks, könnte Lehrkräften dabei helfen, ihr (digitales) Feedback wirksamer zu gestalten.

Wir konnten zudem zeigen, dass die *Fairness* und der *Nutzen* des *wahrgenommenen Feedbacks* wichtige Prädiktoren für das *akademische Selbstkonzept* darstellen. Damit dieser Zusammenhang in der Schule genutzt werden kann, sollte ein Bewusstsein bei den Lehrkräften geschaffen werden. Eine Möglichkeit wäre beispielsweise auch Feedback von Schüler*innen stärker zu nutzen. Es bestehen zudem Unterschiede beim akademischen Selbstkonzept in Bezug auf den sozioökonomischen Status. Wissenschaft und Schule sollten deshalb neue digitale Fördermöglichkeiten zur Genese des *akademischen Selbstkonzeptes* erproben, um soziale Disparitäten weiter zu verringern.

## Footnotes

1 Wir haben in der vorliegenden Studie zusätzlich nach dem Kommunikationsmittel gefragt: *Ich bin mit meinen Lehrkräften in Kontakt über …* Die Ergebnisse zeigten, dass an den Gymnasien signifikant (*p* < 0,01) häufiger E‑Mails (67 %), Schuleigene Websites (46 %) und Zoom (21 %) zum Einsatz kamen. An den anderen Schulformen wurden Microsoft Teams (37 %), WhatsApp (20 %) und das Telefon (14 %) signifikant häufiger verwendet. (Mehrfachantworten waren möglich).
